# Sarcoidosis and Cancer: A Complex Relationship

**DOI:** 10.3389/fmed.2020.594118

**Published:** 2020-11-24

**Authors:** Thomas El Jammal, Michel Pavic, Mathieu Gerfaud-Valentin, Yvan Jamilloux, Pascal Sève

**Affiliations:** ^1^Internal Medicine Department, La Croix-Rousse Hospital, Lyon, France; ^2^Medicine Department, Sherbrooke University, Sherbrooke, QC, Canada; ^3^INSERM U1111, Center International de Recherche en Infectiologie/International Research Center in Infectiology (CIRI), University Claude-Bernard Lyon 1, Villeurbanne, France; ^4^Pôle IMER, Hospices Civils de Lyon, Lyon, France; ^5^HESPER EA 7425, Lyon University, University Claude-Bernard Lyon 1, Lyon, France

**Keywords:** sarcoidosis, granulomatosis, neoplasia, lymphoma, checkpoint inhibitor

## Abstract

Sarcoidosis is a systemic disease of unknown etiology, characterized by the presence of non-caseating granulomas in various organs, mainly the lungs, and the lymphatic system. Since the individualization of sarcoidosis-lymphoma association by Brincker et al., the relationship between sarcoidosis or granulomatous syndromes and malignancies has been clarified through observational studies worldwide. Two recent meta-analyses showed an increased risk of neoplasia in sarcoidosis. The granulomatosis can also reveal malignancy, either solid or hematological, defining paraneoplastic sarcoidosis. Recent cancer immunotherapies, including immune checkpoint inhibitors (targeting PD-1, PD-L1, or CTLA-4) and BRAF or MEK inhibitors were also reported as possible inducers of sarcoidosis-like reactions. Sarcoidosis and neoplasia, especially lymphoma, can show overlapping presentations, thus making the diagnosis and treatment harder to deal with. There are currently no formal recommendations to guide the differential diagnosis workup between the evolution of lymphoma or a solid cancer and a granulomatous reaction associated with neoplasia. Thus, in atypical presentations (e.g., deeply impaired condition, compressive lymphadenopathy, atypical localization, unexplained worsening lymphadenopathy, or splenomegaly), and treatment-resistant disease, targeted biopsies on suspect localizations with histological examination could help the clinician to differentiate neoplasia from sarcoidosis. Pathological diagnosis could sometimes be challenging since very few tumor cells may be surrounded by massive granulomatous reaction. The sensitization of currently available diagnostic tools should improve the diagnostic accuracy, such as the use of more “cancer-specific” radioactive tracers coupled with Positron Emission Tomography scan.

## Introduction

Sarcoidosis is a systemic disease of unknown etiology characterized by multiple granuloma formation in various sites, especially in the lungs, lymph nodes, liver, eyes, and skin (1). Although its etiology is still unknown; sarcoidosis is thought to be the consequence of an exaggerated immune response to an environmental trigger in a genetically predisposed patient. Mortality in sarcoidosis is mainly represented by respiratory failure due to pulmonary fibrosis, central nervous system involvement, and cardiac damage (2, 3). In a French epidemiological study of 2417 patients, the cause of death was linked to neoplasms in 1.8% of the patients. Most of the time, the cause was a solid neoplasm (1.4%) while hematological malignancies represented 0.4% of the deaths in this series (4).

The occurrence of sarcoidosis or sarcoidosis-like reaction (SLR) in cancer patients has been known for several years, either through case reports or larger series (5–8). A localized granulomatous reaction can be found in draining lymph nodes of a solid tumor or in distant sites (mostly spleen, liver, or bone marrow) (6). The granulomatous reactions can also be found at the primary site of the tumor itself. Brincker reported that 3–7% of primary tumor sites may present with epithelioid cell granulomas (9). An SLR can also be found in the case of opportunistic infections in such a population (e.g., cryptococcosis, atypical mycobacteria, tuberculosis, nocardiosis, actinomycosis, …), in case of disseminated *Bacille de Calmette de Guérin* (BCG) infection after instillation of BCG therapy for bladder cancer, or in case of cancer-specific treatments [anti-programmed death-(ligand)1 (PD-(L)1)/anti-cytotoxic T-lymphocyte antigen 4 (CTLA4)/anti-MAP/ERK kinase (MEK)/anti-B-Raf proto-oncogene (BRAF)] (10). Moreover, typical sarcoidosis can occur in solid or hematological malignancies before, during, or after the onset of the disease. In those situations, the diagnosis may be challenging and requires a careful diagnostic workup. Herein, we summarize the specifics for sarcoidosis or SLR mimicking cancer, especially regarding positive and differential diagnosis of sarcoidosis or cancer in this particular association. We provide a brief literature review performed through the PubMed platform (https://www.pubmed.ncbi.nlm.nih.gov) using the keywords “sarcoidosis,” “cancer,” “lymphoma,” “sarcoidosis-lymphoma syndrome”, “sarcoid-like reaction” and “drug-induced sarcoidosis” that allowed us to find most of the references used to build this article.

## Interactions Between Sarcoidosis and Cancer

### Cancer Risk in Sarcoidosis Patients

In previous series and cohorts, patients with sarcoidosis were found to have a higher risk of cancer compared to the general population, especially lymphoma (Table 1). Brincker et al. first reported an increased risk of cancer in sarcoidosis patients (7). Indeed, in this series, the risk of lymphoma was 11 times higher in the sarcoidosis group, compared to the expected risk of lymphoma in the general population. The risk of developing lung cancer was 3 times higher than the one expected in the general population. In the following years, several epidemiological studies analyzed the risk of cancer in sarcoidosis patients, with contradictory data according to the different types of cancer (lymphoma, testicular cancer, digestive cancers, breast cancer, etc.) (5, 8, 13, 17, 21, 23). Of note, Ungprasert et al. reported no increased risk of malignancy in a cohort study of patients with sarcoidosis compared with non-sarcoidosis patients but an increased risk of hematological malignancies in patients with sarcoidosis and extra thoracic involvement compared with those without extra thoracic involvement (26). An increased risk of cancer was noted (e.g., by 30–40%), especially skin cancers, hematological malignancies and leukemias. Despite conflicting data, the overall cancer risk in sarcoidosis patients is clearly higher than in the general population. Indeed, in two recent meta-analyses, the relative risk of developing cancer in patients with sarcoidosis was near 1.19–1.21 [with significant results in both studies (*p* < 0.05)] and the risk of developing hematological malignancies was even higher [RR = 1.92, 95% CI (1.41–2.62)] (27, 28). Lymphomas and particularly Hodgkin lymphomas (HL) were significantly more incident in sarcoidosis [RR = 2.91, 95% CI (1.21–6.98)] (28). There was also an increased incidence of skin cancers [RR = 2.00, 95% CI (1.69–2.36)] and especially non-melanoma skin cancers [RR = 2.29, 95% CI (1.88–2.78)]. These meta-analyses reported no increase in the risk of developing lung cancer. This interesting result is probably explained by a lower prevalence of smokers among sarcoidosis patients compared to the general population (29). Worth noting, there was no specific subgroup analysis of patients with sarcoidosis-related pulmonary fibrosis.

**Table 1:** Risk of malignancy in sarcoidosis patients: cohort and case control studies.

**References**	**Population**	**Relative risk of neoplasia**
Anderson and Engels (11)	418 patients with HCL (case control, United States)	Patients with HCL were more likely to have a sarcoidosis antecedent (OR = 9.6 95% CI = 2.4–39.5)
Askling et al. (5)	474 sarcoidosis patients (cohort study in Sweden)	No increased risk of lymphoma, increased risk of melanoma (SIR = 1.6 95% CI = 1.0–2.3), and non-melanoma skin cancer (SIR = 2.8 95% CI = 2.0–3.8)
Blank et al. (12)	435 sarcoidosis patients (cohort)	Possible association (incidence 14%)
Boffetta et al. (13)	5,768 sarcoidosis patients (case-control study in USA)	No increased risk of malignancy (globally)
Brincker (14)	17 patients (case series) in Denmark	Increased risk of lymphoma
Brincker (15)	131 patients (case review)	Increased risk of lymphoma
Hemminki et al. (16)	5,149 sarcoidosis patients (cohort in Sweden)—female cancers	No global increased risk of malignancy
Ji et al. (17)	10,037 sarcoidosis patients (cohort study in Germany)	Increased risk of malignancy (SIR = 1.40) and cancer diagnosed later than 1 year of follow-up (SIR = 1.18). Increased risk of non-melanoma skin cancer, kidney and non-thyroid endocrine tumors, non-Hodgkin lymphoma, and leukemia
Kataoka et al. (18)	148 sarcoidosis patients (cohort)	Increased risk of leukemia, thyroid, and larynx cancer
Kristinsson et al. (19)	16 sarcoidosis patients in HL patients (case control study in Sweden)	Increased risk of having a sarcoidosis in patients with HL (OR = 3.7 95% CI = 1.9–7.4)
Landgren et al. (20)	7,476 patients (case control study in Sweden and Denmark)	Increased risk of lymphoma (OR = 14.1 95% CI = 5.4–36.8)
Le Jeune et al. (21)	1,153 sarcoidosis (case control study in UK)	Increased risk of malignancy (RR = 1.65 95% CI = 1.22–2.24) and especially skin cancer (RR = 1.86 95% CI = 1.11–3.11)
Mellemkjaer et al. (22)	50 sarcoidosis patients (case control study in Denmark)	Increased risk of NHL (OR = 1.9 95% CI = 1.3–2.7)
Rømer et al. (8)	555 sarcoidosis patients (cohort in Denmark)	No increased risk of malignancy (O/E ratio = 1.16 95% CI = 0.75–1.79)
Seersholm et al. (23)	254 sarcoidosis patients (cohort in Denmark)	No increased risk of malignancy (SIR = 1.4 95% CI = 0.99–2.0)
Smedby et al. (24)	3,055 patients with NHL Sweden and Denmark (case control)	No global increased risk of lymphoma in sarcoidosis patients
Søgaard et al. (25)	12,890 sarcoidosis patients (cohort study in Denmark)	Global increased risk of malignancy (SIR = 1.3 95% CI = 1.3–1.4), increased risk of lung cancer, tonsil cancer, and lymphoma. Of note, lung cancer risk seems to be more important in the first 3 months and then substantially decrease
Ungprasert et al. (26)	345 sarcoidosis patients (case control study in the USA)	No global increased risk of malignancy, but higher risk of hematological malignancies in patients with sarcoidosis and extra thoracic involvement (HR = 1.87 95% CI = 1.09–3.22)

*CI, confidence interval; HCL, hairy cell leukemia; HR, hazard ratio; NHL, non-Hodgkin lymphoma; O/E, observed/expected; OR, odd ratio; RR, rate ratio; SIR, standardized incidence ratio; UK, United Kingdom; USA, United States of America*.

In a study about mortality in sarcoidosis patients in France, Jamilloux et al. reported that non-Hodgkin lymphoma was the most frequently declared cause of death in women when sarcoidosis was not the underlying cause of death, especially after the age of 50 (4). In another recent review of 115 cases, thyroid and breast cancers were the most frequently reported solid neoplasms (30).

### Sarcoidosis and Sarcoid-Like Reactions in the Course of Cancer

Sarcoidosis and SLR can occur before, during or after cancer (31). While sarcoidosis is a well-defined condition, SLR is usually defined as non-caseating granulomatous reaction occurring under various conditions, which do not meet the diagnostic criteria for sarcoidosis (32). Many alternative diagnoses mimicking sarcoidosis can also be encountered in a neoplastic context (Table 2). In a series of 29 sarcoidosis patients with pre-existing cancer, Arish et al. described clinical and radiological features of granulomatosis (55). Histological features were not described in this article. Breast cancer and lymphoma were the most commonly observed malignancies. Sarcoidosis was frequently diagnosed at an early stage, possibly due to a more systematic follow-up with computed tomography (CT) and positron emission tomography in cancer patients. Radiological features were similar to those seen in classical sarcoidosis (mediastinal and hilar lymphadenopathy). Most patients were asymptomatic at the sarcoidosis diagnosis. The patients had bronchoalveolar fluid (BALF) lymphocytosis and granuloma on endobronchial biopsies and parenchymal biopsies, suggesting a pattern of systemic immune response rather than a local granulomatous response to neoplastic cells. In 43% of patients, the diagnosis of sarcoidosis was made more than 5 years after the diagnosis of cancer. De Charry et al. described the characteristics of granulomatosis occurring in the context of lymphoma (56). In this study, the patients developed granulomatosis at a median age of 60 while typical sarcoidosis usually occurs before 50 with a peak of incidence between 20 and 39 (1). Sarcoidosis explained 4 of the 25 patients' granulomatous manifestations in this study. Other etiologies were hematological malignancies (*n* = 11), tuberculosis (*n* = 3), allergy (*n* = 1), disseminated annular granuloma (*n* = 1), atypical inflammatory bowel disease (*n* = 1), and undetermined granulomatosis (*n* = 4). Likewise, London et al. described a series of 39 patients with sarcoidosis occurring in the setting of lymphoma (57). The median age at the onset of sarcoidosis was 49 years. Most patients had a history of high stage lymphoma (Ann Harbor III or IV) (74%). Most patients developed sarcoidosis after terminating lymphoma chemotherapy and all except two were considered in complete remission. In another series, Herron et al. reported that almost 60% of sarcoidosis cases occurring after a cancer were diagnosed within 1 year of cancer diagnosis (58).

**Table 2:** Non-exhaustive list of differential diagnosis of granulomatosis in pre-existing cancer patients.

**Type of granulomatosis**	**Etiology**	**Risk factors**
Opportunistic infectious granulomatosis (bacteria)	Tuberculosis	Immunosuppression [e.g., chemotherapy, hematological malignancy, …; (33)]
	Atypical mycobacteria	Immunosuppression [e.g., chemotherapy, hematological malignancies, …; (34)]
	Disseminated BCG infection (superficial bladder cancer)	BCG therapy for bladder cancer treatment could lead to disseminated BCG infection (35)
	Nocardiosis	Immunosuppression [e.g., hematological malignancies, diabetes, …; (36, 37)]
	Actinomycosis	Immunosuppression (38)
Opportunistic infectious granulomatosis (fungi)	Cryptococcosis	Immunosuppression [e.g., diabetes, cirrhosis, CD4 lymphopenia, stem cell transplant, chemotherapy, …; (39)]
	Candida spp.	Immunosuppression (chemotherapy) (40)
	Aspergillosis	Immunosuppression (41)
	Pneumocystosis	Immunosuppression [e.g., solid or hematological malignancies; (42)]
Opportunistic infectious granulomatosis (parasites)	Disseminated strongyloidosis	Immunosuppression [e.g., hematological malignancies; (43)]
	Toxoplasmosis	Immunosuppression [e.g., solid or hematological malignancies; (44)]
Opportunistic infectious granulomatosis (viruses)	HCV ++	HCV associated liver cancer or HCV associated lymphoma (45)
	HBV +/–	HBV associated liver cancer/hepatic granuloma (46)
	EBV	Lethal midline granuloma (47), lymphomatoid granulomatosis (48)
	CMV	Doughnut granuloma (immunosuppression) (49)
Drug-induced granulomatosis	ICI (nivolumab, pembrolizumab, cemiplimab, avelumab, durvalumab, ipilimumab)	Treatment of lung cancer, pharyngolaryngeal cancer, melanoma, renal cancer (10)
	BRAF/MEK inhibitors (dabrafenib, vemurafenib, trametinib, cobimetinib)	Treatment of lung cancer, melanoma (10)
	IFN-α	Formerly used in melanoma, kidney cancer, lymphoma (50)
	BCG therapy	Used in bladder cancer (35)
Histologic granuloma associated to neoplasia	Lymphoma (Hodgkin and non-Hodgkin lymphoma)	Proper to neoplasia (6)
	Solid neoplasia (testicular cancer, melanoma, breast cancer)	Proper to neoplasia (32)
	Immune restauration	Following aplasia (immune reconstitution leads to granuloma associated with T cell infiltrate) (51)
	Donor-acquired sarcoidosis	Following HSCT (52)
	Lymphomatoid granulomatosis	Proper to neoplasia (48)
Primary immunodeficiencies associated with neoplasia (especially lymphoma)	Common variable immunodeficiency, GATA2 mutations (Mono MAC syndrome)	Primary immunodeficiency related granulomatosis (53, 54)

*BCG, Bacille de Calmette et Guérin; CMV, cytomegalovirus; EBV, Epstein Barr Virus; GATA2, GATA binding protein 2; HBV, hepatitis B virus; HCV, hepatitis C virus; HSCT, hematopoietic stem cell transplant; ICI, immune checkpoint inhibitors; IFN-α, interferon alpha; MAC, Mycobacterium avium complex*.

During the course of cancer, an epithelioid granuloma can be found in regional lymph nodes or in distant metastases (6). Some authors have suggested that the presence of a cancer-associated SLR could be a marker of good prognosis, indicating a strong immune response to tumor cells (59–62). This type of reaction is mainly seen in lymphoma and testicular cancer (6, 63, 64). Other authors have provided conflicting data regarding other types of cancers, especially non-small lung carcinoma in which the presence of granulomas was not associated with better prognosis (65, 66).

Some cancer treatments can also induce granuloma formation. The description of various side effects, including sarcoidosis, has come with the recent advent of immune checkpoint inhibitors (ICI), as well as BRAF/MEK inhibitors. SLR were described either with ICI [anti-PD1: pembrolizumab (67), nivolumab (68); anti-PD-L1: atezolizumab (69), durvalumab (70), avelumab (71); anti-CTLA4: ipilimumab (72)] or with BRAF/MEK inhibitors [vemurafenib (73), dabrafenib (74) sometimes in combination with trametinib or cobimetinib (75)]. A review of the WHO pharmacovigilance database including 2425 drug-induced sarcoidosis was conducted in 2019. In this study, strong associations were found between SLR and several drugs including, pembrolizumab, nivolumab, ipilimumab (*n* = 103) along with dabrafenib, vemurafenib, trametinib, and cobimetinib (*n* = 37) (76). SLR disappeared with drug discontinuation in 17.7% of the cases. In a few patients, drug reintroduction triggered SLR recurrence. Stronger associations were found with other drugs such as tumor necrosis factor alpha inhibitors (TNFi) and especially soluble TNF receptor etanercept, interferon and PEG-interferon. These SLR can also mimic cancer progression or metastases. In a series of 45 patients treated with ICI, 10 developed SLR and 2 developed mediastinohilar lymphadenopathy misinterpreted as metastatic progression (77). Sarcoidosis and SLR have to be considered as differential diagnosis in patients with such treatments and biopsies have to be performed since no radiological nor biological marker is sufficiently specific to assess sarcoidosis diagnosis especially when presentation is suspicious [e.g., asymmetric lymph node enlargement; (78)]. The Society for Immunotherapy of Cancer Toxicity Management Working Group has made recommendations regarding immunotherapy-related SLR (79, 80). Corticosteroids (CS) have similar indications to those of “idiopathic” sarcoidosis. Pulmonary function testing and chest CT have to be performed in order to assess sarcoidosis severity. In case of DLCO decrease >20%, total lung capacity >10% or forced vital capacity >15%, persistent sarcoidosis-related symptoms, radiographic progression, or involvement of critical extrapulmonary organ systems or sarcoidosis related hypercalcemia, it is recommended to hold the treatment with ICI and add CS at 1 mg/kg/day dosage. CS tapering and withdrawal will depend on the clinical response.

Sarcoidosis has also been reported in hematopoietic stem cell transplant recipients. It was described either in allogenic (81–85) or autologous bone marrow transplantation (86, 87). In these cases, sarcoidosis was described in various organs such as lymph nodes (85), liver (88), lung, or lymph nodes (82). In some cases, a specific condition called “donor-acquired sarcoidosis” was described (52, 82, 84, 85). This condition refers to the occurrence of sarcoidosis in a solid organ or allogenic bone marrow transplant recipient when sarcoidosis is previously known in the donor.

In the past decade, TNFi were found to be an efficient way to treat sarcoidosis patients (89–92). The immunosuppressants can be linked to a theoretical increased risk of malignancy. However, in 2012, Maneiro et al. reported an incidence rate of cancer of 1 per 100 patients-year in sarcoidosis patients treated with TNFi (93). In a recent review, Adler et al. reported data from randomized and non-randomized clinical trials of TNFi in sarcoidosis patients. The malignancies occurred in <1% of the patients (94). In comparison with other inflammatory diseases, TNFi does not seem to increase the risk of cancer in sarcoidosis (95).

### The Sarcoidosis-Lymphoma Syndrome

Sarcoidosis-lymphoma association was first described by Brincker in a series of 46 patients (14). In this series, sarcoidosis-lymphoma syndrome was defined as a condition in which sarcoidosis occurred several years before the diagnosis of lymphoma. Most frequently the diagnosis of sarcoidosis was made after 40 years old and the most frequent type of lymphoma was HL. On the contrary, Papanikolaou and Sharma have found that NHL were the most common lymphomas. Interestingly, the development of new lymphadenopathy or new splenic involvement were the main symptoms revealing lymphoma in this series. These patients were on average 10 years older at the sarcoidosis diagnosis compared to unselected patients in most series (96, 97). Compared to the general population, sarcoidosis patients had a 5.5-fold higher risk of developing lymphoma (14). In a recent monocentric study of patients with sarcoidosis-lymphoma syndrome compared to unselected sarcoidosis patients, significant differences between initial or follow-up patients' characteristics have been evidenced especially regarding angiotensin-converting enzyme (ACE) blood levels that have proved to be higher in sarcoidosis-lymphoma syndrome, while the sarcoidosis alone group was more likely to have lung involvement, a restrictive ventilatory defect and a higher relapse rate (98).

Most of the time, lymphoma occurs 2–8 years after the sarcoidosis diagnosis, preferentially in patients with a chronic course of the disease (14). CD4/CD8 lymphocyte ratio in BALF is also higher in patients with sarcoidosis-lymphoma syndrome compared to unselected patients (98). For example, B-cell activating factor (BAFF) levels are elevated in patients with sarcoidosis and are correlated with ACE levels (99). Elevation of pro-proliferative cytokines such as BAFF for B lymphocytes could be a possible explanation for the emergence of clonal proliferation in sarcoidosis patients in comparison with other autoimmune diseases (100).

## When Should We Look For Neoplasia in Patients With Sarcoidosis?

Sarcoidosis diagnosis requires three major conditions: (1) a compatible clinical/radiological presentation, (2) evidence of granulomas on a biopsy sample, and (3) exclusion of differential diagnoses (101).

Although rarely observed, physicians should be aware that sarcoidosis can present itself as a pseudo tumoral condition such as miliary nodules, peritoneal involvement, and symptomatic osteolytic or osteoblastic lesions (102–104).

Other red flags should alert the clinician about the atypical nature of sarcoidosis or the possibility of underlying neoplasia [e.g., impaired general condition, compressive phenomena, hemoptysis, refractory disease; (31, 32, 105)]. Atypical radiological manifestations should also be considered. For example, unilateral, compressive or necrotizing lymph nodes are not usually seen during the course of sarcoidosis. Isolated mediastinal lymphadenopathy without hilar lymph node enlargement, non-lymphatic diffuse lung micronodules, cavitary mass on chest X-ray should also be considered as suspicious for a differential diagnosis of sarcoidosis (106, 107). Broadly speaking, these atypical presentations should encourage the clinician to pay attention to other causes of granulomatosis, including lymphoma, infectious granulomatosis (tuberculosis, leprosy, syphilis, brucellosis, Q fever, Whipple's disease), common variable immunodeficiency, and drug-induced sarcoidosis (108).

In a patient with previously known sarcoidosis, the occurrence of atypical manifestations (e.g., peritoneal or gut involvement) or new organ involvement, and refractory disease which is defined as a disease in which a 2nd line treatment is not sufficient to achieve satisfying disease control or satisfying CS tapering, must lead to histological confirmation to rule out opportunistic infection and lymphoma, especially (31, 102, 108).

Recently, the American thoracic society (ATS) provided new guidelines concerning sarcoidosis diagnosis (32). In a large review of 16 studies enrolling a total of 556 patients with suspected stage I sarcoidosis, 85% of sampling procedures with histological examination confirmed the diagnosis of sarcoidosis. In 11% of the cases, histology was inconclusive, and in 2% of the cases, a differential diagnosis was made. Among differential diagnoses, 25% were lymphoma. On the basis of this work, ATS reminds that the diagnosis of sarcoidosis does not only rely on histological findings but also on compatible presentation and exclusion of differential diagnoses.

As noted above, sarcoidosis patients have a possibly increased risk of malignancy, either solid or hematological. The increase of the risk of developing solid neoplasia in the course of sarcoidosis seems to be less important than the risk of developing hematological malignancies such as lymphoma (7, 19, 20). Again, this emphasizes the attention the clinician should pay to any atypical symptom or presentation in a sarcoidosis patient since delayed diagnosis of cancer may impact the patient's prognosis.

## How to Diagnose Neoplasia in Patients With Sarcoidosis?

A histological examination is warranted to accurately diagnose a patient with sarcoidosis or sarcoid-like reaction to neoplasia. The neoplastic cells can be found on histological examination within a granulomatous reaction. In case of atypical sarcoidosis, lymphocytes phenotyping should be performed in order to rule out clonality. Full examination of included samples (which increases sensibility of histological examination) and complementary immunohistochemical staining could also be helpful. A specific subtype of HL, the necrotic granuloma-like HL, as well as some T-cell lymphoma (NHL) could be misdiagnosed as non-neoplastic granuloma, such as sarcoidosis, because of an important tumor-related sarcoid reaction and only careful histologic examination can help to rectify the diagnosis (109–111). Among the neoplasia which can mimic sarcoidosis, special attention should be paid to lymphomatoid granulomatosis (LYG). LYG is a lymphoproliferative disorder associated with Epstein Barr virus (EBV). The aggressive behavior of the tumor is represented by its metastatic potential. The classic histological pattern of LYG is a coexistence of granulomatous inflammation made of large atypical EBV-positive B cells, T cells, necrosis, and lymphocytic vasculitis (112). Lung localization and skin involvement may mimic sarcoidosis. Almost 100% of patients present with pulmonary involvement consisting most of the time in pulmonary nodules. Skin nodules are also part of the clinical presentation. They take the form of subcutaneous nodules, most of the time erythematous and painful (113). Histological diagnosis is difficult if the pathologist is not aware of the suspected diagnosis of LYG or unfamiliar with this condition. Classical histological results consist of a mononucleated infiltrate with large and small lymphocytes invading vascular walls and a variable amount of atypical CD20+ B cells among numerous small CD3+ T cells.

Although some imaging results may point to a diagnosis of neoplasia [e.g., asymmetric lymphadenopathy, hypermetabolism of extrathoracic lymph nodes; (78)], 18-fluorodeoxyglucose (18-FDG) uptake on PET-CT is unable to differentiate malignant from non-malignant hypermetabolism. Currently, 18-FDG PET-CT may be used to identify the best biopsy sites, which may result in the observation of tumor cells or specific granuloma characteristics which suggest other causes of granulomatosis [e.g., loosely organized collections of phagocytes or multinucleated giant cells, extensive or dirty necrosis, or palisading granulomas; (114, 115)]. Other radiotracers or techniques used with PET-CT may be interesting in differentiating tumoral hypermetabolism from non-malignant hypermetabolism. Dual time point 18-FDG PET-CT with delayed acquisition sequences and 18F-3′-Fluoro-3′-deoxythymidine (18F-FLT) PET-CT could help in distinguishing malignant from non-malignant lesions but few studies are available and their roles in improving the diagnostic performances of PET-CT remain to be precised (116, 117).

Magnetic resonance imaging (MRI) changes could also be helpful in distinguishing sarcoidosis lesions from tumoral localizations. Although conventional MRI is insufficient to distinguish malignant bone lesions from bone sarcoidosis (118), Conte et al. reported one case where the differential diagnosis between sarcoidosis and metastasis was made using whole body diffusion MRI (119). In this patient, the hypersignal on diffusion sequences contrasted with a decreased signal in apparent diffusion coefficient sequences that was considered to be too low to be compatible with neoplastic origin.

Finally, specific biomarkers have been proposed to ease the diagnosis, especially in germ-cell tumors. In such cases, the elevation of serum levels of α-fetoprotein, human chorionic gonadotropin and lactate dehydrogenase may help guide the diagnosis (32). No suggestion has been made regarding other types of cancer, probably due to the lack of specificity of tumor markers in these settings.

## Conclusion

Granulomatosis and cancer can coexist in various clinical situations that the clinician should be aware of. The risk of developing solid neoplasia or hematological malignancies, especially lymphomas, is increased in sarcoidosis patients. Sarcoidosis-lymphoma syndrome has to be considered in patients with previously known sarcoidosis and unexplained recurrence of deep or peripheral lymph nodes enlargement. Any atypical and unexplained symptom mimicking a sarcoidosis flare should encourage the clinician to be careful to differential diagnosis.

The granulomatous reactions are not uncommon in the course of solid neoplasia and hematological malignancies. Recent therapeutic advances in cancer treatment, especially the emergence of immunotherapy with ICI, have reminded the possibility of drug induced SLR as it was previously known with older therapies (e.g., interferon). CS may help control ICI-induced SLR without holding cancer treatments.

Differentiating sarcoidosis from cancer-associated granulomatosis is difficult. Atypical presentation of sarcoidosis (atypical organ involvement or refractory disease) may alert the clinician. There is currently no alternative to the histological examination to differentiate sarcoidosis from neoplasia. New radiotracers (18F-FLT) and new acquisition techniques (dual time point PET CT) are promising but currently not available in routine care.

A careful and rigorous diagnosis process is required when encountering granulomatosis, on the one hand, because of the increased risk of neoplasia in sarcoidosis patients and, on the other hand, because of the sarcoidosis-like presentation of neoplasia. Discussing with the pathologist in order to sensitize the diagnosis (full examination of included samples, complementary immunohistochemical staining, search for clonality) is fundamental.

The sarcoidosis patients are also susceptible to present neoplasia as the general population. Diagnosis can be difficult maybe due to a greater propension to present a granulomatous reaction compared to the general population. Here again, it is essential to share and to discuss clinical presentation to ease the pathologist's work.

## Author Contributions

TE contributed to bibliography, most of writing, and reviewing of the manuscript. PS, YJ, MG-V, and MP contributed to reviewing and writing the manuscript. All authors contributed to the article and approved the submitted version.

## Conflict of Interest

The authors declare that the research was conducted in the absence of any commercial or financial relationships that could be construed as a potential conflict of interest.
